# Intrauterine Ischemic Reperfusion Switches the Fetal Transcriptional Pattern from HIF-1α- to P53-Dependent Regulation in the Murine Brain

**DOI:** 10.1371/journal.pone.0110577

**Published:** 2014-10-17

**Authors:** Yupeng Dong, Takuya Ito, Clarissa Velayo, Takafumi Sato, Keita Iida, Miyuki Endo, Kiyoe Funamoto, Naoaki Sato, Nobuo Yaegashi, Yoshitaka Kimura

**Affiliations:** 1 Advanced Interdisciplinary Biomedical Engineering, Tohoku University Graduate School of Medicine, Sendai, Japan; 2 Department of Obstetrics & Gynecology, Tohoku University Hospital, Sendai, Japan; National University of Singapore, Singapore

## Abstract

Ischemic reperfusion (IR) during the perinatal period is a known causative factor of fetal brain damage. So far, both morphologic and histologic evidence has shown that fetal brain damage can be observed only several hours to days after an IR insult has occurred. Therefore, to prevent fetal brain damage under these circumstances, a more detailed understanding of the underlying molecular mechanisms involved during an acute response to IR is necessary. In the present work, pregnant mice were exposed to IR on day 18 of gestation by clipping one side of the maternal uterine horn. Simultaneous fetal electrocardiography was performed during the procedure to verify that conditions resulting in fetal brain damage were met. Fetal brain sampling within 30 minutes after IR insult revealed molecular evidence that a fetal response was indeed triggered in the form of inhibition of the Akt-mTOR-S6 synthesis pathway. Interestingly, significant changes in mRNA levels for both HIF-1*α* and p53 were apparent and gene regulation patterns were observed to switch from a HIF-1*α*-dependent to a p53-dependent process. Moreover, pre-treatment with pifithrin-*α*, a p53 inhibitor, inhibited protein synthesis almost completely, revealing the possibility of preventing fetal brain damage by prophylactic pifithrin-*α* treatment.

## Introduction

During the late stages of development, HIF-1*α* is known to play a central role in brain development, along with down-regulation of both P53 protein and mRNA [1]–[3] around the same time. HIF-1*α* and P53 have established antagonistic responses to Ischemic reperfusion (IR) and their overall balance gives us a glimpse of the underlying ongoing homeostatic process [4]–[7].

Berger and Ganier have attributed fetal ischemic reperfusion to intrauterine asphyxia during the perinatal period [8], [9]. In the course of IR, mediators such as hypoxanthine, oxygen free radicals and cytokines are observed to accumulate [10]–[13], resulting in fetal brain injuries such as hypoxia ischemic encephalopathy and cerebral palsy [14]. It may take several hours to days before IR induced brain damage can be microscopically evident while most acute responses to IR occur at the molecular level through the production of pro-inflammatory cytokines [15]. An example of which is the appearance of *IL-1β* mRNA within 15 minutes of cerebral ischemia [8], [9].

The response to IR, resulting in most cerebral ischemic reperfusion injuries, occurs via two main signaling pathways: (1) the phosphorylation of JNK which induces pro-cell death; and (2) the inhibition of Akt phosphorylation which down regulates cell survival [16]. Phosphorylated JNK regulates gene expression through downstream targets such as P53, whereas activation of P53 regulates Akt pathway by feedback inhibition [17]. P53 dependent cell death plays an important role during various types of brain damage such as subarachnoid hemorrhage [18], [19]. In this particular IR response, P53 dependent cell death was revealed by not only increased p53 target gene expression, but also enhanced protein-protein interaction between P53 and other proteins [20]–[22]. Significantly, pretreatment with PFT-*α*, a p53 transcriptional activity inhibitor, was observed to reduce ischemic brain injury in mice [23], [24].

## Materials and Methods

All animal experiments in this study were conducted in accordance with the Tohoku University guidelines for animal experimentation. All the experimental protocols in this study were approved by the Tohoku University Committee for Safety Management of Animals.

### Mice and samples

C57BL/6N mice were used for all experiments. Mice were purchased from CLEA Japan Inc. (Japan). Mice weighing 18–22 g (9–14 weeks) were bred overnight marking gestation day 0 (GD 0) under specific pathogen-free conditions at the Animal Research Institute of Tohoku University. For blocking transcriptional activity of P53 [23], Pifithrin (PFT)-*α* (sc222176, SANTA CRUZ) was administered by a single subcutaneous (s.c.) injection done between 3 to 6 hours prior to sampling on day 18 of gestation (GD18) respectively.

Fetal brain and heart sampling were aided by microscopy on GD18 after IR treatment (with a 30-minute mean time from the end of last release to the last sample collection). Tissues were flash-frozen in liquid nitrogen and stored at −80 degrees Celsius.

### Intrauterine IR

**Figure 1:**
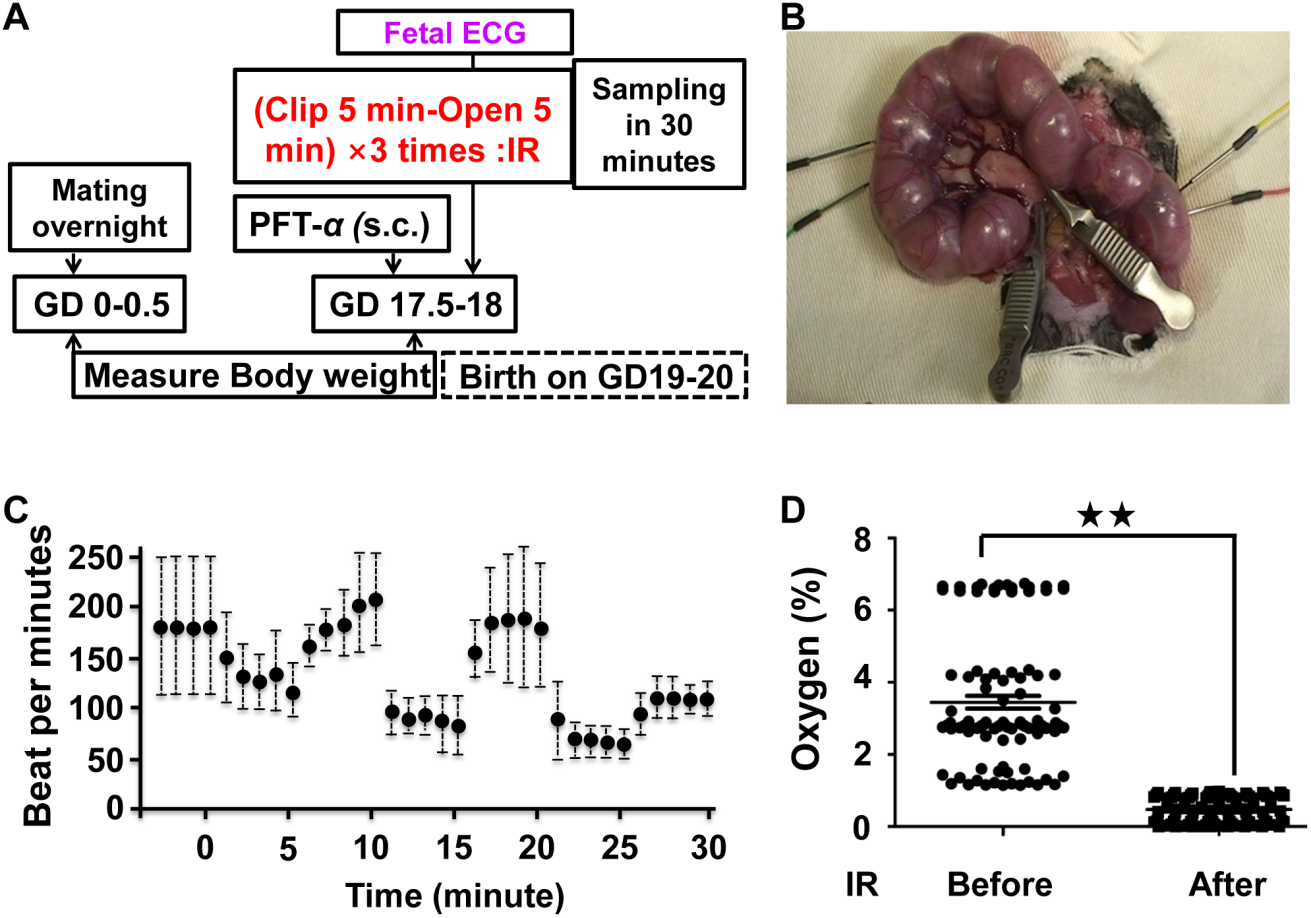
An ischemic reperfusion in pregnant mice. (A) Experimental design (B) No IR treatment pregnant mice and Ischemic reperfusion (IR) mice underwent surgery on day 18 of gestation. (C). Fetal electrocardiography (FECG) was used to monitor the conditions of both the mother and the fetuses in either clipped or non-clipped uterine horns (*n* = 5 fetuses from at least 5 individual pregnant mice). (C) Measurement of oxygen concentration in the amniotic fluid before and after IR (*p*<0.01 indicated as two stars, *n* = 5 fetuses from at least 3 individual pregnant mice).

Aseptic conditions were maintained all throughout the experiment. Pregnant mice were anesthesized with subcutaneous ketamine (Ketalar 500 mg Daiichi-Sankyo: 100 mg/Kg) and xylazine (ROMPUN INJ. SOLUTION 2% Bayer; 10 mg/Kg) and maintained with inhalational isoflurane (Forane AbbVie Inc.: 0.5%, 260 ml/min). Laparotomy was done to expose both uterine horns. After a 2-minute waiting period for heart rate conditions to stabilize, the uterine artery and ovarian artery were clipped (Figure 1A). A clip-release cycle consisted of 5 minutes of clipping followed by 5 minutes of release. A total of 3 clip-release cycles were undertaken during which a fetus from the clipped horn and a fetus from the non-clipped horn were continuously monitored using fetal electrocardiography (FECG) [25]–[27]. FECG in the International Patent Application Number: PCT/JP2006/316386 and Clip-release cycles in the Japanese Patent Application Number 2009/176683. In mice, reassuring fetal status was suggested by a fetal heart rate variability ranging between 120–250 beats per minute (bpm). Fetal heart rate (FHR) was noted to vary according to the fetal number. IR decreased the FHR of fetuses beginning at 30 seconds from clipping. During the second occlusion, the FHR of fetuses decreased to a minimum of 60 bpm and this was reversible after the second release. In the last release of the third cycle, complete reversal to normal occurred within 5 minutes (near 100 bpm) but this could not be achieved without significant changes in the heart rates of the mother or of the fetus of the non-clipped side. The establishment of intrauterine IR per experiment was confirmed through this data (Figure 1B).

Temperature and humidity were controlled at 36.5±1°C and 65±10%, respectively, during the surgery.

Oxygen concentrations of amniotic fluid derived from the clip side of IR mice before and after IR treatment were measured using a micro fiber optic oxygen meter (Microx TX3, TAITEC).

### Antibodies and Reagents

#### Western blot

Total extracts from each fetal brain or heart were incubated with 1% NP40 RIPA buffer (Cell Signaling Technology) on ice for 30 minutes followed by complete homogenization. After centrifugation at 14,000 rpm for 15 minutes, the supernatant containing 30 µg protein was measured using a BCA kit (23227, Thermo Scientific).

All primary Antibodies were used for western blot at 1∶1000 and incubated for overnight at 4 degree. Antibodies were purchased from Novus Biologicals (NB100-134, Anti-HIF-1*α* antibody), Abcam (ab58530, Anti-MDM2) and Cell Signaling Technology: Anti-Actin (5125), anti-P53 (2524), anti-phosphorylation of P53 (S15) (9284), anti-acetylation of P53 at Lys379/Lys382 (2570), anti-TLR4 (2219), anti-ERK1/2 (4695) at Thr202/Tyr204, anti-phosphorylation of ERK1/2 (4376), anti-P38 (9212), anti-phosphorylation of P38 at Thr180/Tyr182 (9211), anti-SAPK/JNK (9258), and anti-phosphorylation of SAMP/JNK at Thr183/Tyr185 (4668), anti-phosphorylation of AKT at ser473 (4060), anti-phosphorylation of mTOR at Ser2448 (5536), anti-PARP (9532).

### Analysis of Gene Expression (DNA Microarray)

Total mRNA extracted from 12 fetal brains from each treatment group were utilized. A total of 6 Toray 3D-Gene Mouse Oligo chip 24 K (Toray Industries, Inc., Tokyo, Japan) microarrays were analyzed per treatment. Each chip utilized a 0.5 µg portion of combined total RNA from a matched pair of male and female samples. RNA was amplified and labeled using an Amino Allyl MessageAmp II RNA Amplification kit (Life Technologies Japan Ltd.) according to the manufacturer’s instructions. Each sample of RNA was labeled with fluorescence Cy3 or Cy5 and cohybridized at 37°C for 16 hours. These were subsequently washed and dried. Hybridization signals were scanned using Scan Array Express (Perkin Elmer, MA, USA) and global background analysis was performed using GenePx Pro (MDS Analytical Technologies, CA, USA). All 36 arrays were then normalized together as one experiment to reduce nonbiological variability. *Mapk*, *Atf2*, *P53* and *Hif-1α gene expression* were analyzed. Housekeeping gene *Hprt* was used as a standard control.

### Chromatin immunoprecipitation (ChIP) assay

To confirm genes regulated by HIF-1*α* and P53, fetal brains were collected from different mouse dams on GD18. According to the User Guide of EpiQuik Tissue Chromatin Immunoprecipitation Kit (P-2003), the fetal brain was washed using 1 ml of 1x PBS(−) and fragmented by pipetting in a 1.5 ml centrifugal tube. This was then added to 0.9 ml of crosslink solution (final concentration of 1% from 16% formaldehyde) and incubated at room temperature for 20 minutes while under constant rotation. Afterwards, 100 ul of 1.25 M glycine solution was added prior to centrifugation at 800 rpm for 5 minutes. The tissue samples were then resuspended in 200 ul CP3 prior to sonication (5 second on and 5 second off for 1 minute at power 30%).

For ChIP assay, antibody for 1∶50 HIF-1 (Novus Biologicals, NB100-134) and for 1∶200 p53 (2524S, Cell signaling Technology) were used. To design the all Chip-qPCR primers, first, the binding position and the binding sequence for transcriptional factors HIF-1alpha or p53 were searched by using 2 databases: Search ChIP-qPCR Assay-SABiosciences (QIAGEN) and UCSC In-Silico PCR. Next, Chip-qPCR primers were designed by Primer3Plus software. The sequences used as primers of specific gene promoters have been provided as Supporting Information S1.

### Statistical Analysis

Western blot data were analyzed using Image J software. Graphs were made and analyzed using Graphpad Prism 5J software. Parameters derived were expressed in mean ± standard deviation (SD). The statistical method used was the Student’s *t* test (two tailed for independent samples) with a probability (*p*) of less than 0.05 considered as significant.

## Results

### Experiment design

A simple mouse experiment protocol was used to induce the intrauterine IR (Figure 1, materials and methods) during late stage pregnancy and prior to birth at day 18 of gestation. In this present work, molecular confirmation revealed the acute response to IR in fetal brains. A total of 66 fetuses from 25 mouse dams were used for analysis. Successful IR was confirmed by FECG in the clipped side (Figure 1B, 1C and Supporting Information S2). In addition, Oxygen concentration in the amniotic fluid decreased to almost 0% after IR treatment as compared to levels before IR treatment (Fig. 1D).

### JNK1/2 was activated as a response to intrauterine IR treatment

**Figure 2:**
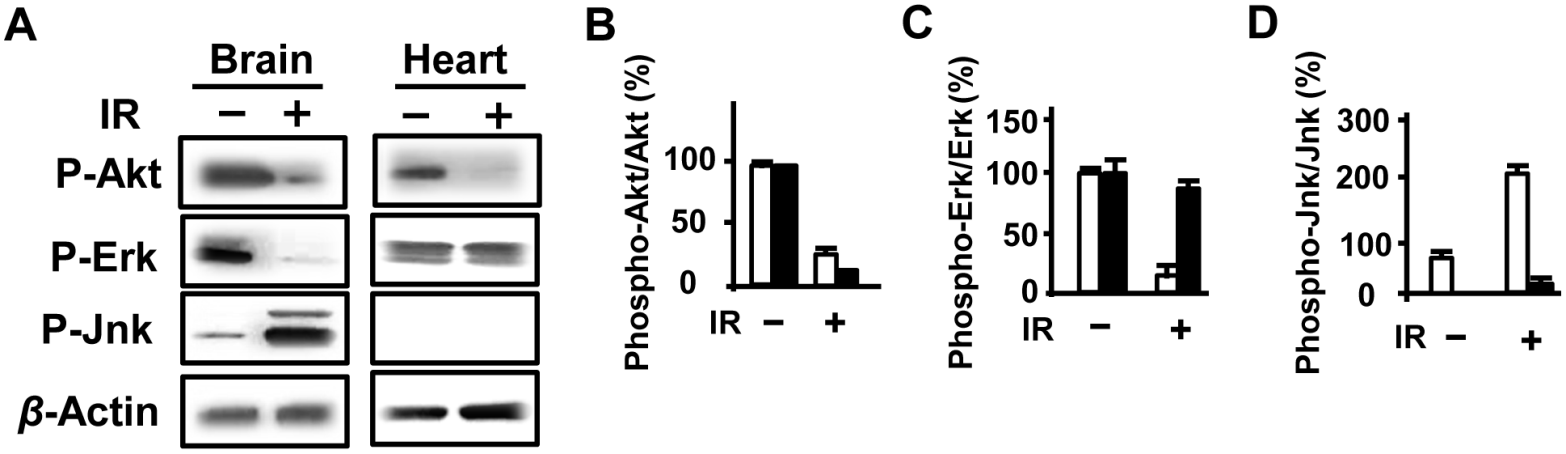
IR changed signaling pathways in the fetal brain. (A) The phosphorylation of JNK1/2 was activated in the fetal brain without (−) or with IR (+). The phosphorylation of both Akt and ERK1/2 were suppressed in the fetal brain by IR. (B, C, D) Measurement and Graph data *p*<0.01 indicated as two stars (*n*  = 5 fetuses from at least 3 individual pregnant mice). White means fetal brain and black means fetal heart.

To understand the immediate response to IR, various cell-signaling pathways were investigated. With preliminary experiments, significant changes appeared in MAPKs by IR in the fetal brain compared with no significant change in the fetal heart. Phosphorylation of ERK1/2 at Thr202/Tyr204 was suppressed while the phosphorylation of JNK1/2 at Thr183/Tyr185 was increased by IR (Fig. 2). Moreover, phosphorylation of JNK1/2 critically increased within 30 minutes and was time course-dependent in its increase after IR. In addition, there was no phosphorylation of p38 at Thr180/Tyr182 in both fetal brain and heart.

### Protein synthesis inhibition as a response to intrauterine IR

**Figure 3:**
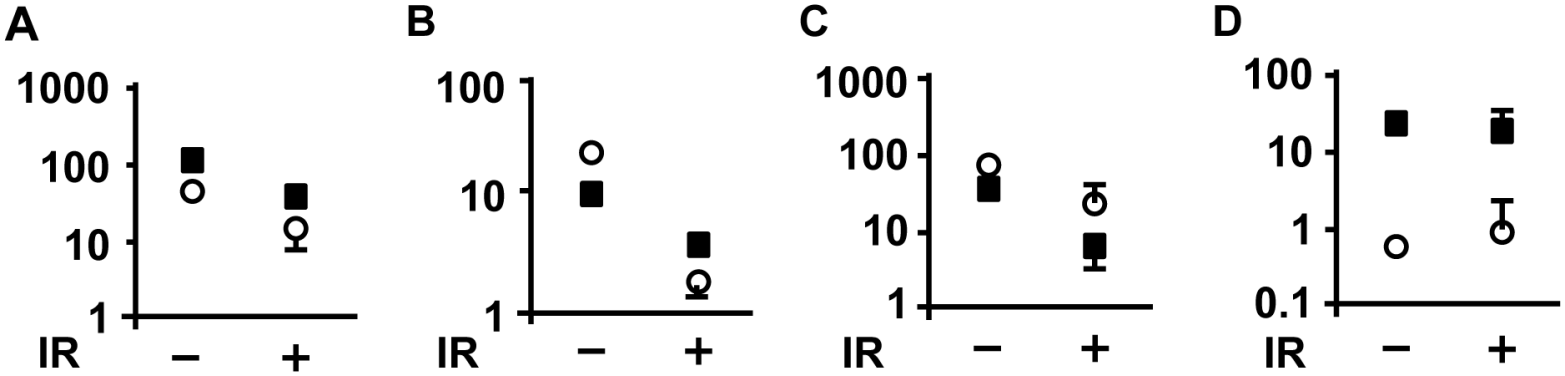
IR inhibited protein synthesis in the fetal brain. (A) Phosphorylation of Akt, (B) Phosphorylation of mTOR, (C) Phosphorylation of S6, and (D) Cleaved-Parp of either fetal brain or heart was measured. (*n* = 4 fetuses from at least 3 individual pregnant mice). White means fetal brain and black means fetal heart.

Phosphorylation of Akt, mTOR and S6 were used as protein synthesis markers while cleaved-Parp was used as an apoptosis marker in the present work [28]. Our Western blot analysis data implied that protein synthesis was suppressed by IR accompanied by high levels of apoptosis in the fetal brain (Figure 3). There were low levels of apoptosis in the fetal heart in contrast to high levels of apoptosis in the fetal brain in either N or IR group (Figure 3D).

### Not Hif-1 α but p53 was increased in response to IR

**Figure 4:**
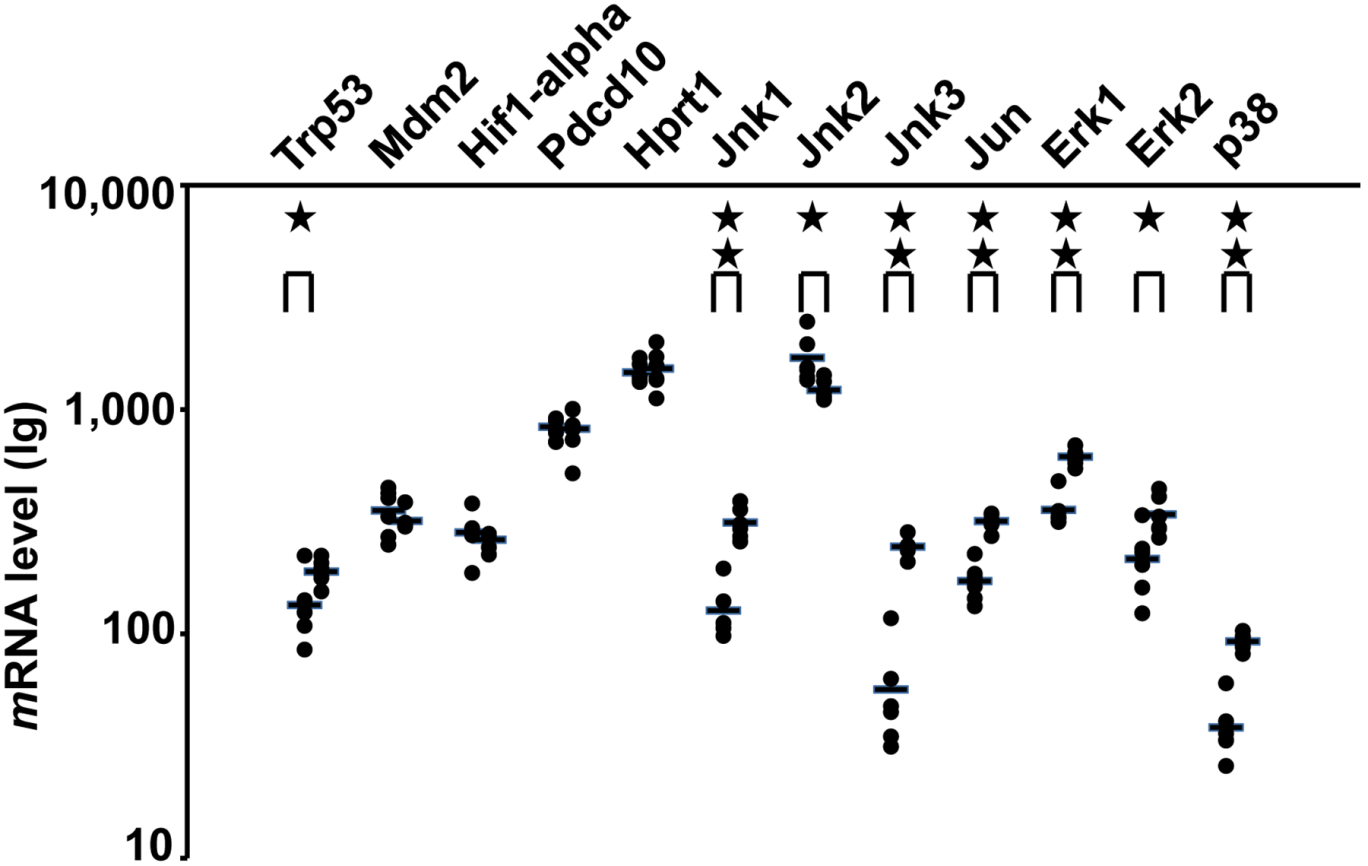
P53 mRNA was increased response to IR in the fetal brain. Within 30 minutes post-IR, the mRNA levels of *Mapk associated proteins,* except *Jnk2* and *Trp53* (*p53*), were noted to have increased, while *Hif-1α, Mdm2* and *Ccm3/Pdcd10* were not increased as compared to the housekeeping gene *Hprt1*. (*n* = 12 from at least 3 individual pregnant mice). *p*<0.05 indicated as single star and *p*<0.01 indicated as two stars.

To investigate new protein synthesis after IR, full gene expression in fetal brain was evaluated by microarray analysis (Figure 4). Within 30 minutes after IR, the expressions of *Mapk* genes were increased except for *Jnk2*. Within 30 minutes after IR, the mRNA level of *p53* was noted to have been increased, while *Hif-1α* was not increased as compared to the housekeeping gene *Hprt1*.

**Figure 5:**
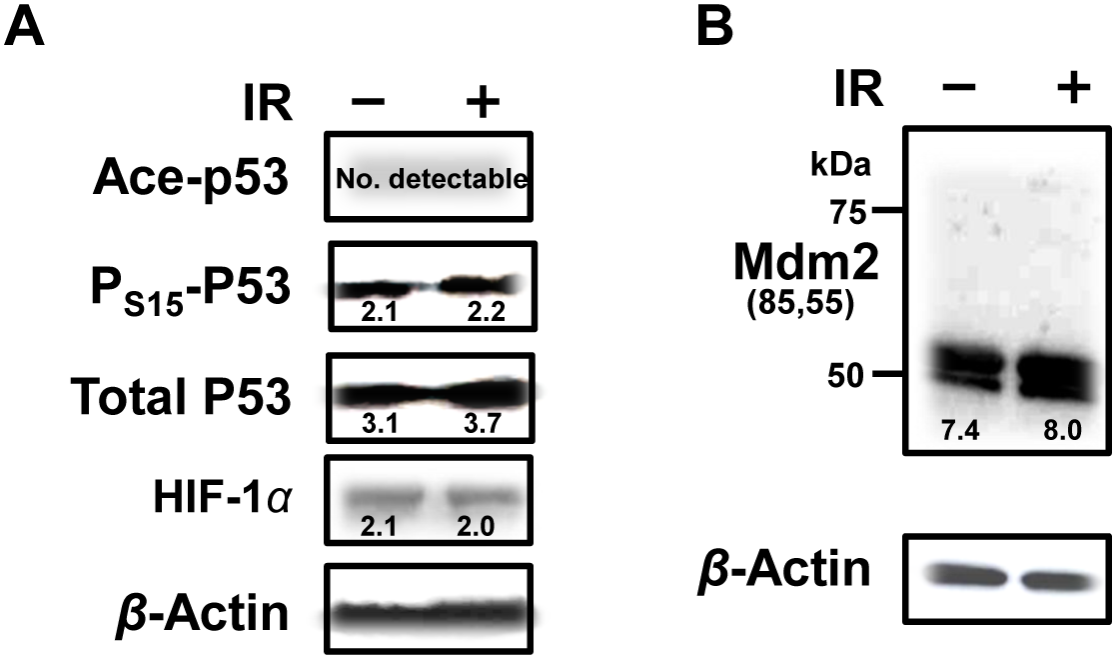
P53 was increased response to IR in the fetal brain. Even though no significant change in protein level, total p53 and phosphorylation of P53 at ser15 were increased compared with HIF-1*α* in fetal brains response to IR. Numbers in the pictures show the average of three independent experiments (*n* = 3 from 3 individual pregnant mice).

On the other hand, Even though no significant change in protein levels for both HIF-1*α* and P53 were observed within the 30 minutes after IR, either phosphorylation of p53 or total P53 and mdm2 either phosphorylation of p53 or total P53 and mdm2 might start to increase compared with HIF-1*α* (Figure 5).

### P53 and not Hif1- α banded onto gene promoters in response to IR

**Figure 6:**
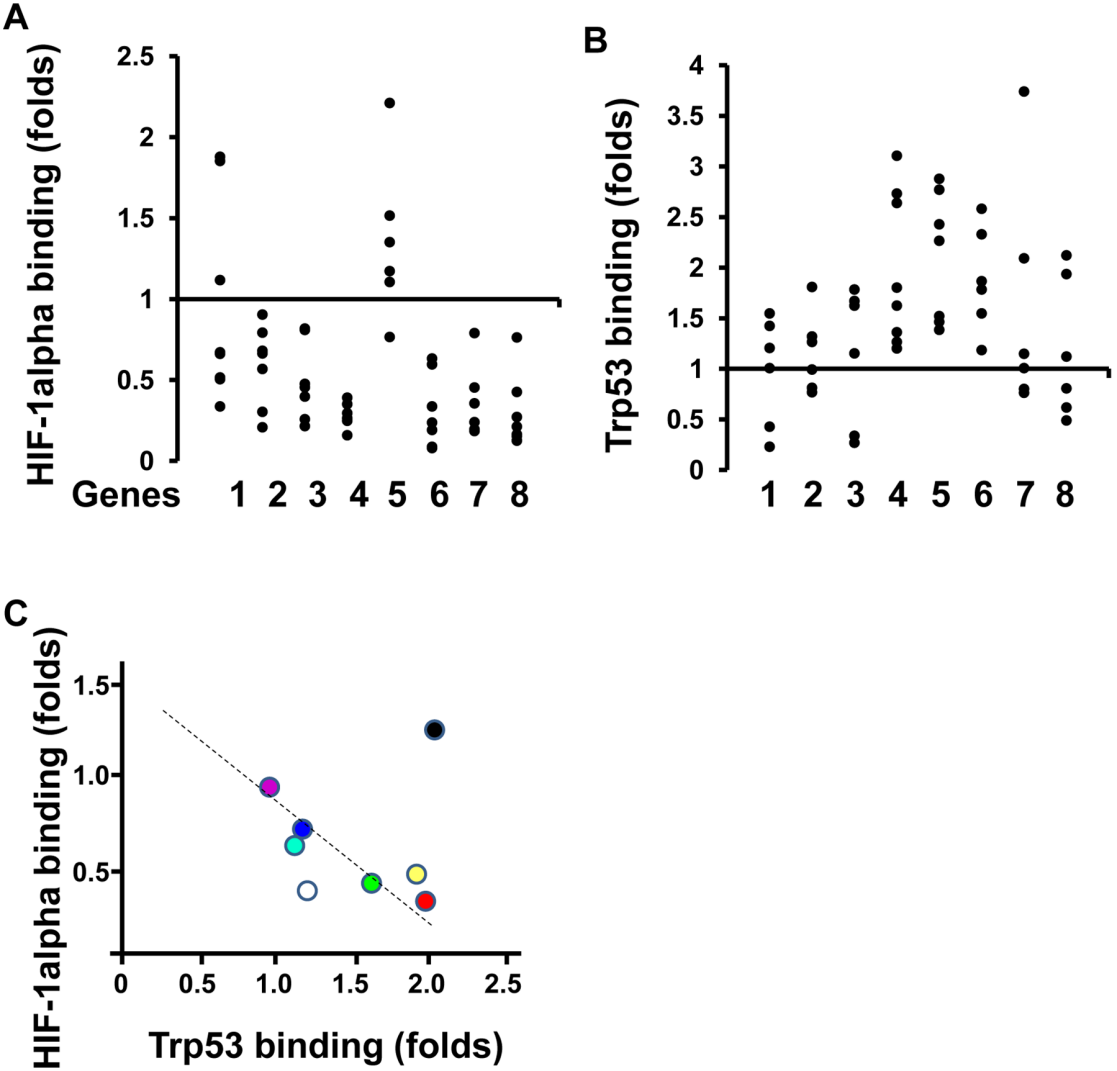
Transcriptional pattern was changed in the fetal brain. Binding to gene promoters by two transcriptional factors, HIF-1*α* (A) and P53 (B), was measured by ChIP assay. Fold change from 0.5 to 2 was considered as not significant. Gene No. 1.P53; 2.Mdm2; 3.Akt1; 4.Pdcd10; 5.Pfkfb4; 6.S100A10; 7.Cox4i1; 8.Sirt1. Binding Folds =  (After IR)/(Before IR) (*n* = 6∼8 from at least 3 individual pregnant mice, each dot means one sample). (C) HIF-1*α* release and P53 binding on the promoter of indicated genes. From left to right according the number of x-axis, they are trp53 (purple), Akt1 (sky blue), mdm2 (blue), Sirt1 (White), cox4i1 (green), s100A10 (yellow), pdcd10 (red) and pfkfb4 (black).

To understand the transcriptional activity of Hif-1*α* and p53 after IR, chromatin precipitation assay was done. In the fetal brain after IR, a decrease in Hif1-*α* binding to promoter sites of genes was noted in contrast to P53 which showed a significant increase (Figure 6A, 6B). Thus, there was a negative correlation with HIF-1*α* and a positive correlation with P53 (Figure 6C).

### PFT-α inhibited the phosphorylation of S6

**Figure 7:**
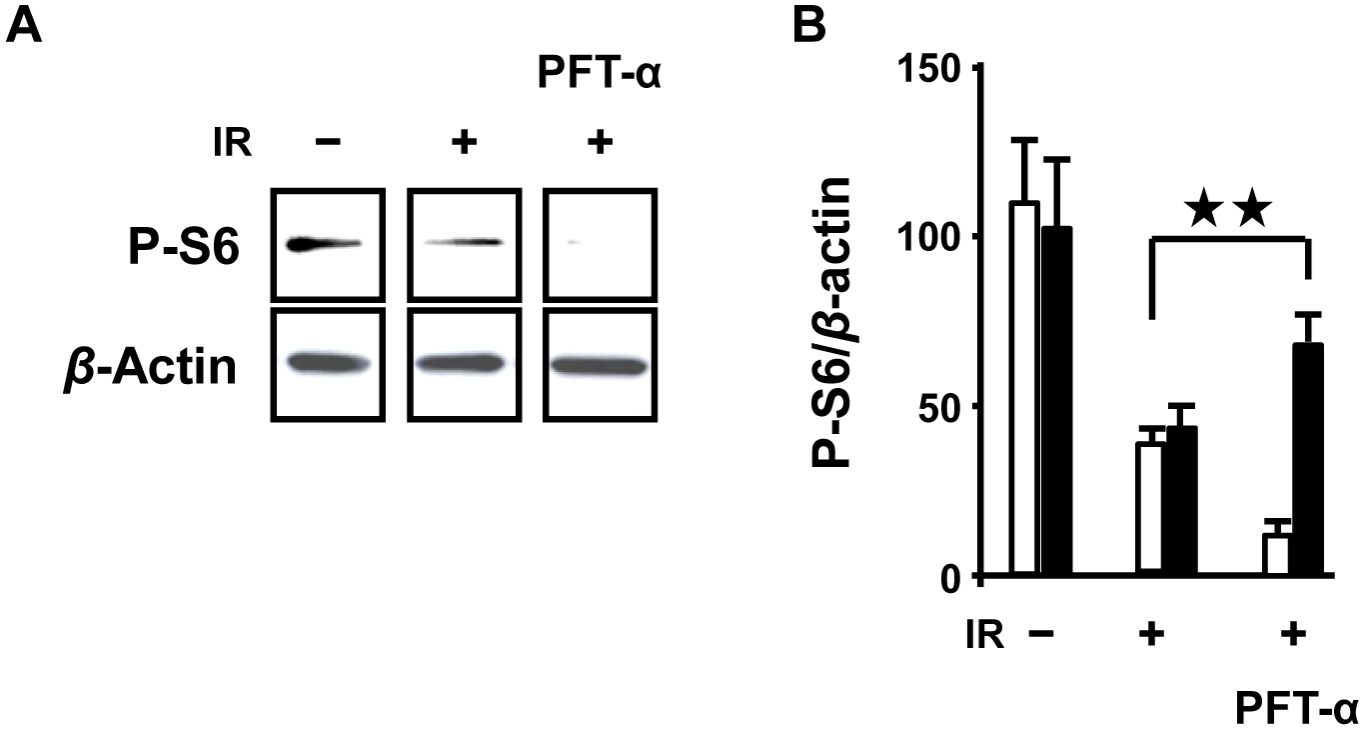
*In vivo*, PFT-*α* inhibition of protein synthesis after IR. (A) PFT-*α* inhibited phosphorylation of S6 in fetal brain. (B) Measurement phosphorylated S6 (*Vs.* beta actin, *n* = 4∼6 from at least 3 individual pregnant mice). White means fetal brain and black means fetal heart.

To confirm whether P53 dependent cell death such as apoptosis was activated after IR, we studied the phosphorylation of S6. IR suppressed the phosphorylation of S6 and this was further suppressed by a p53 inhibitor, PFT-α (Figure 7). The action of PFT-*α,* a mitochondrial p53 inhibitor, in inhibiting P53 protein and transcriptional activity was consistent with previous reports [23], [24]. Overall, our findings suggested that P53 dependent protein synthesis was activated by IR.

## Discussion

### Transcriptional pattern changes secondary to IR

In the present study, we focused on the acute response to IR. During our window of within 30 minutes from the initial hypoxemic insult, fetal brain damage was not microscopically evident. The ability to evaluate acute brain responses during the early stages post-IR may be the key to understanding the development of fetal brain damage. The prevailing view is that p53 induces cell death after IR leading to fetal brain damage. Our study focused on phosphorylation of JNK, its’ downstream target P53 and protein synthesis to evaluate fetal brain damage. Other causes such as necrosis and autophagy are beyond the scope of this study.

A summary of our findings is as follows: on gestation day 18, gene expression was regulated by HIF-*α* not by P53 for brain development [1]–[3]. As soon as within 30 minutes after IR, phosphorylation of JNK was activated resulting in p53 binding onto gene promoters (Figure 2 and 6). Specifically, the pattern of transcriptional regulation by HIF-1*α*/P53 changed from HIF-1*α*-dependent to P53-dependent in response to IR (Figure 6).

On the other hand, after exposure to intrauterine IR, phosphorylation of Akt, mTOR and S6 were observed to be suppressed (Figure 3). This data suggested that protein synthesis was inhibited although resulting apoptotic activity remained high the in fetal brain (Figure 3). The remaining uninhibited activity consisted of new protein synthesis via p53 regulated gene expression response to IR (Figure 6).

Moreover, PFT-α completely inhibited the phosphorylation of S6 suggesting that protein synthesis dependent on P53 was not suppressed within 30 minutes post-IR (Figure 7). This revealed the role of IR in changing cellular transcriptional patterns from a HIF-1α dependent manner to a P53 dependent manner.

Our study suggests that potential fetal brain damage caused by IR could be naturally prevented (Supporting Information S3) and would entail concomitant Mdm2 accumulation (Figure 5B). Accumulated mdm2 may serve to inhibit p53 accumulation at a later stage of post-IR which may prevent fetal brain damage, because Akt promotes mdm2 translocation into the nuclei [29]. However, during the acute stage of post-IR phosphorylation, Akt was evidently inhibited (Figure 2). This supported our assumption that p53 accumulation occurred within 30 minutes post-IR (Figure 5A).

### PFT-*α* decreased vulnerability to fetal brain damage

PFT-*α* is a recently proposed neuroprotectant [30], [31] although, for fetuses in utero, the neuroprotective properties of PFT-*α* remain unclear. The present work implies that the action of PFT-*α* is to block p53 transcriptional activity at an early stage. PFT-*α* reduced P53 dependent protein synthesis which resulted in cell death and related processes such as apoptosis. In several animal experiments involving pre-treatment with PFT-*α* prior to the induction of a subarachnoid hemorrhage (SAH), reports have shown that cerebral vasospasm was reduced [18], [32], [33]. Thus pretreatment with PFT-*α* may serve to rescue fetuses from cerebral hemorrhage.

### Conclusion

When intrauterine IR occurs in maternal mouse dams, it immediately changes the pattern of gene expression by switching on or off specifically paired antagonists, the transcriptional factors HIF-1*α* and P53, in the GD18 fetal brain. Furthermore, inhibition of both P53 dependent transcription and protein synthesis by pre-treatment with PFT-*α* may rescue fetuses from IR induced brain damage.

## Supporting Information

Supporting Information S1 **ChIP assay primers list.** 22–23 bp length of Chip-qPCR primers were designed by Primer3Plus software (in Materials and Methods).
(TIF)

Supporting Information S2 **Raw data using fetal electrocardiograph (FECG).** Form the top to the bottom of each picture, there were ECG of mother mice, Clip side (IR) fetus and Non-clip side fetus. (A) before IR, (B) ischemia, Only IR side fetus (middle) has shown a decreased bpm. (C) reperfusion.
(TIF)

Supporting Information S3 **Phosphorylation of S6 in a timely manner of changes after IR in the fetal brain.** Phosphorylation of S6 was significantly increased at 3, 6 hours after IR.
(TIF)
