# Supplementary figures and images for: Intrauterine Ischemic Reperfusion Switches the Fetal Transcriptional Pattern from HIF-1α- to P53-Dependent Regulation in the Murine Brain
Source: PLoS One. 2014 Oct 17;9(10):e110577. doi: 10.1371/journal.pone.0110577 (PMC4201554; doi:10.1371/journal.pone.0110577)

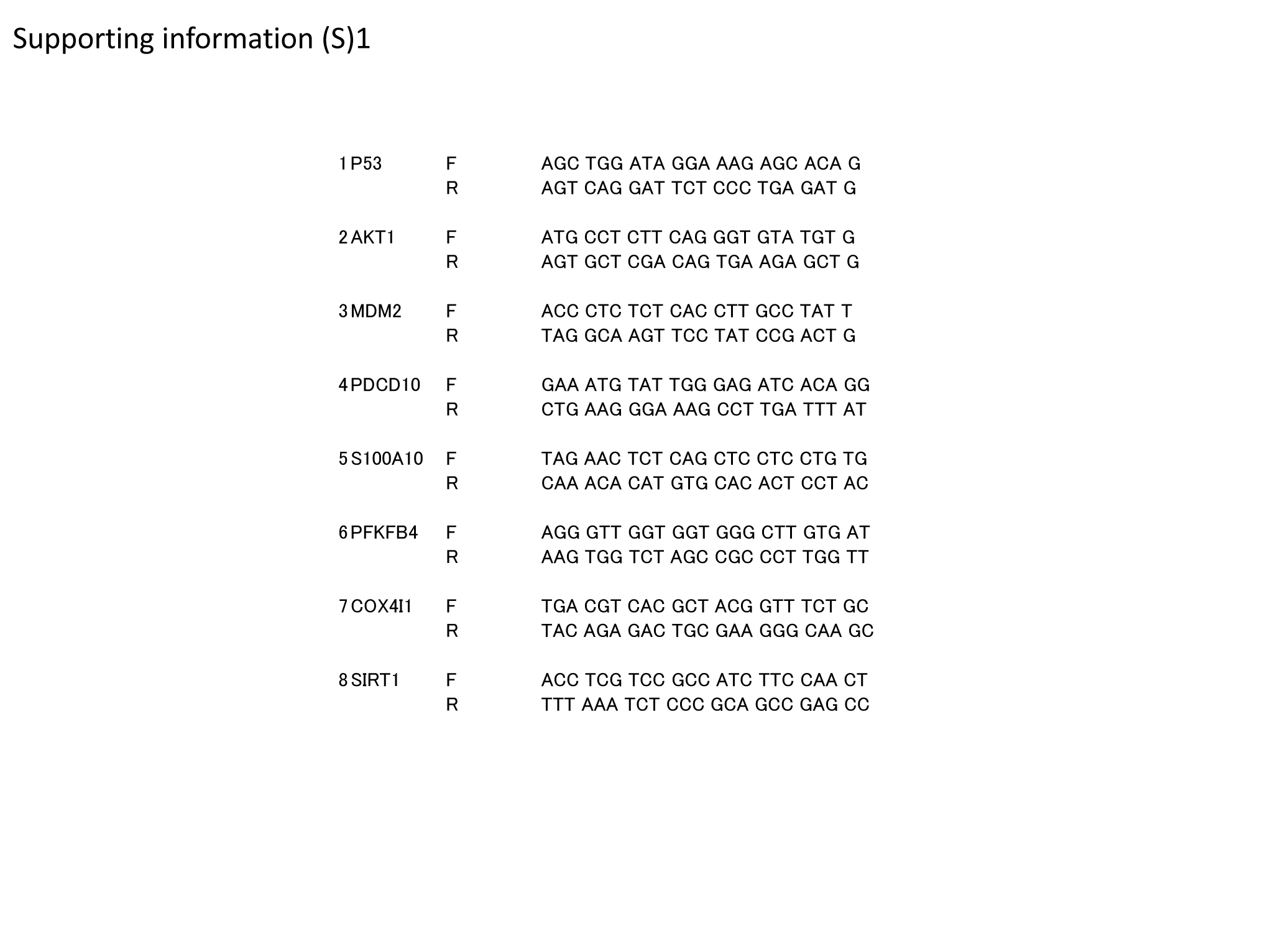

Supplement: Supporting Information S1 — ChIP assay primers list. 22–23 bp length of Chip-qPCR primers were designed by Primer3Plus software (in Materials and Methods). (TIF) [file pone.0110577.s001.tif]

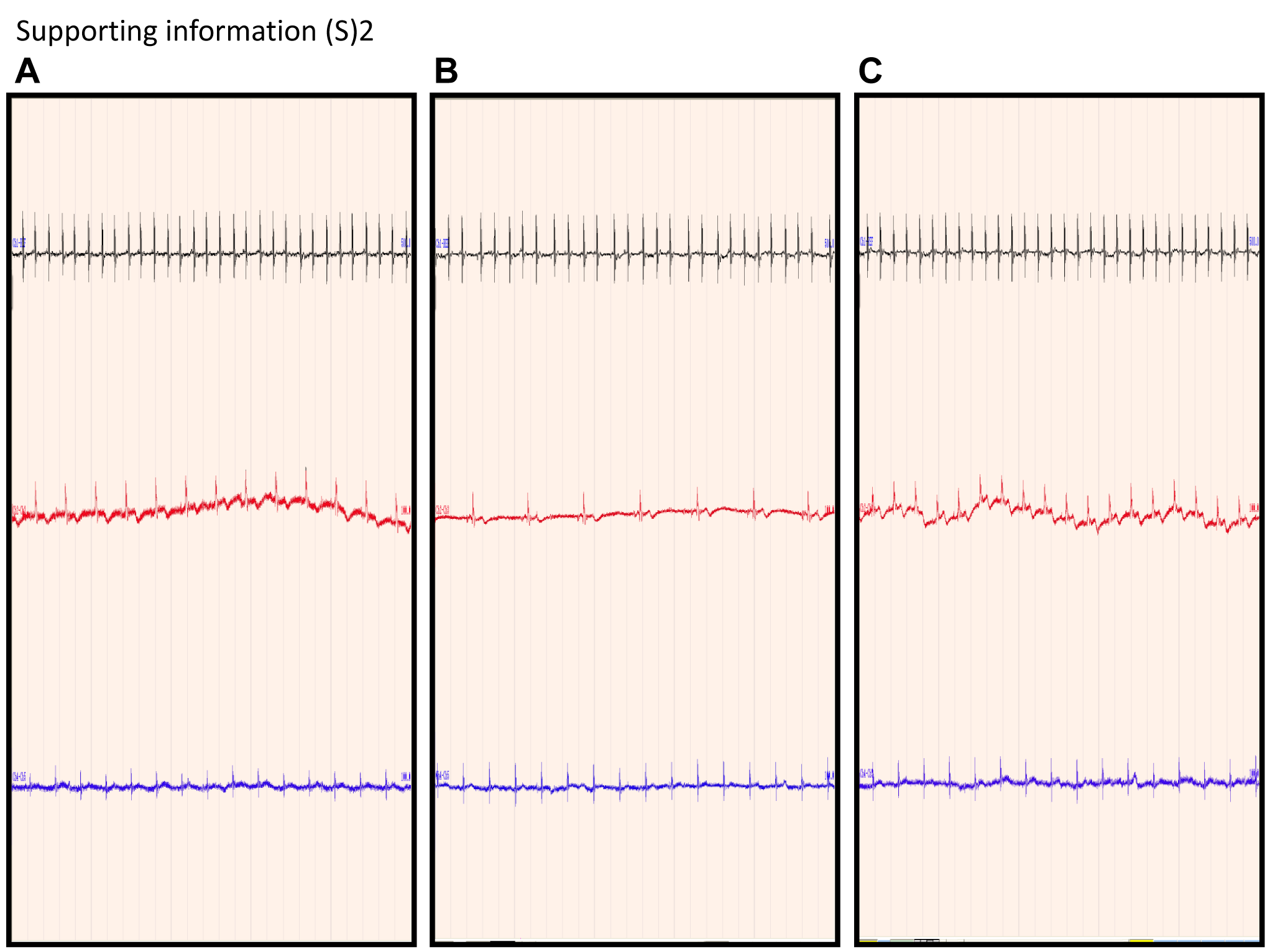

Supplement: Supporting Information S2 — Raw data using fetal electrocardiograph (FECG). Form the top to the bottom of each picture, there were ECG of mother mice, Clip side (IR) fetus and Non-clip side fetus. (A) before IR, (B) ischemia, Only IR side fetus (middle) has shown a decreased bpm. (C) reperfusion. (TIF) [file pone.0110577.s002.tif]

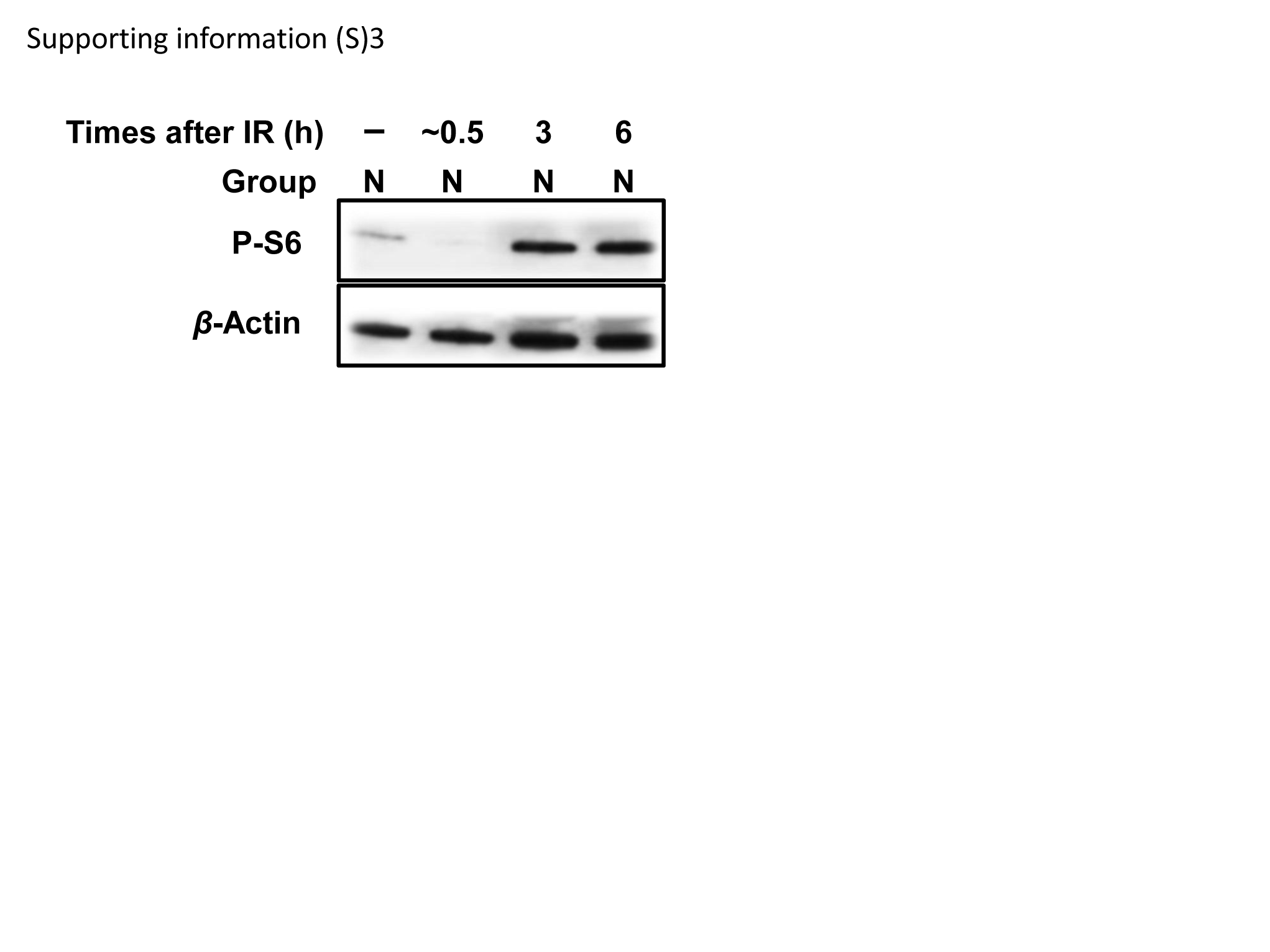

Supplement: Supporting Information S3 — Phosphorylation of S6 in a timely manner of changes after IR in the fetal brain. Phosphorylation of S6 was significantly increased at 3, 6 hours after IR. (TIF) [file pone.0110577.s003.tif]
